# Augmented reality for intracranial meningioma resection: a mini-review

**DOI:** 10.3389/fneur.2023.1269014

**Published:** 2023-11-02

**Authors:** Diego F. Gómez Amarillo, Edgar G. Ordóñez-Rubiano, Andrés D. Ramírez-Sanabria, Luisa F. Figueredo, María P. Vargas-Osorio, Juan F. Ramon, Juan A. Mejia, Fernando Hakim

**Affiliations:** ^1^Department of Neurosurgery, Hospital Universitario Fundación Santa Fe de Bogotá, Bogotá, Colombia; ^2^Department of Neurological Surgery, Fundación Universitaria de Ciencias de la Salud (FUCS), Hospital de San José – Sociedad de Cirugía de Bogotá, Bogotá, Colombia; ^3^Department of Neurosurgery, Clínica del Occidente, Bogotá, Colombia; ^4^Healthy Brain Aging and Sleep Center (HBASC), Department of Psychiatry at NYU Langone School of Medicine, New York, NY, United States

**Keywords:** neurosurgery, meningioma, augmented reality, computer-mediated reality, neuronavigation

## Abstract

Augmented reality (AR) integrates computer-generated content and real-world scenarios. Artificial intelligence's continuous development has allowed AR to be integrated into medicine. Neurosurgery has progressively introduced image-guided technologies. Integration of AR into the operating room has permitted a new perception of neurosurgical diseases, not only for neurosurgical planning, patient positioning, and incision design but also for intraoperative maneuvering and identification of critical neurovascular structures and tumor boundaries. Implementing AR, virtual reality, and mixed reality has introduced neurosurgeons into a new era of artificial interfaces. Meningiomas are the most frequent primary benign tumors commonly related to paramount neurovascular structures and bone landmarks. Integration of preoperative 3D reconstructions used for surgical planning into AR can now be inserted into the microsurgical field, injecting information into head-up displays and microscopes with integrated head-up displays, aiming to guide neurosurgeons intraoperatively to prevent potential injuries. This manuscript aims to provide a mini-review of the usage of AR for intracranial meningioma resection.

## 1. Introduction

Augmented reality (AR) has been defined as adding virtual components within the real world (1). An AR system may provide virtual augmentation of vision, hearing, olfaction, or gustation (2). The most remarkable aspect of AR is influencing mental mapping to generate higher knowledge and improve decision-making processes (2). It is paramount to differentiate AR from virtual reality (VR) and mixed reality (MR). VR is defined as a technology or tool that allows exploration and manipulation of computer-generated artificial or real 3D multimedia sensory environments in real-time (3), while MR merges VR and AR, merging real and virtual worlds (4). Meningioma surgery relies on achieving maximal resection while improving neurological deficits and quality of life whenever possible. Many efforts have been made to improve surgical outcomes to accomplish these goals. In the last three decades, advancements in different technologies, including image-guided systems and other intraoperative monitoring tools, have allowed safer procedures (5, 6). The rapid evolution of neuronavigation systems has also demonstrated the capacity of computer-assisted technology to provide neurosurgeons with a wide range of information, including neurovascular relationships, tumor boundaries, and many others that, combined, may help to improve intraoperative decisions. Some selected cases may be more favored, like reoperations or those cases with anatomical variations (7), where the identification of arteries and nerves becomes more challenging. The implementation of AR has been gaining place given the rising availability of microscopes with integrated head-up displays (HUDs) as well as the current research on head-mounted displays (HMDs).

In the past few years, AR has been demonstrated to be helpful for resecting both brain (8) and skull base tumors (7). The most important feature that makes AR advantageous is its capacity to provide an excellent anatomical understanding of the pathology and surrounding structures (8). AR has been proposed for meningioma surgery, aiming to facilitate surgical orientation, especially for skull base meningiomas (9). The following indications have been used for AR in intracranial meningiomas: invasive tumors with encasement of the internal carotid artery and the middle cerebral artery, tumors in close relationship to the optic chiasm, giant tumors (>10 cc), or recurrent tumors (9). Even though a few case series (9, 10) and case reports (11, 12), have shown the benefits of this technology for meningioma resection. This mini-review briefly enhances the most relevant information in the usage of AR for meningioma resection. Highlighting how the integration of AR into the operating room has permitted a new perception of neurosurgical diseases, not only for neurosurgical planning, patient positioning, and incision design but also for intraoperative maneuvering and identification of critical neurovascular structures and tumor boundaries. It also resumes current limitations and research gaps given the rapid advances in this topic and the constant new information of AR applied for meningioma surgery. Also, potential remarkable developments in the field are discussed.

## 2. Review

### 2.1. Microscope-base AR setup

**Figure 1:**
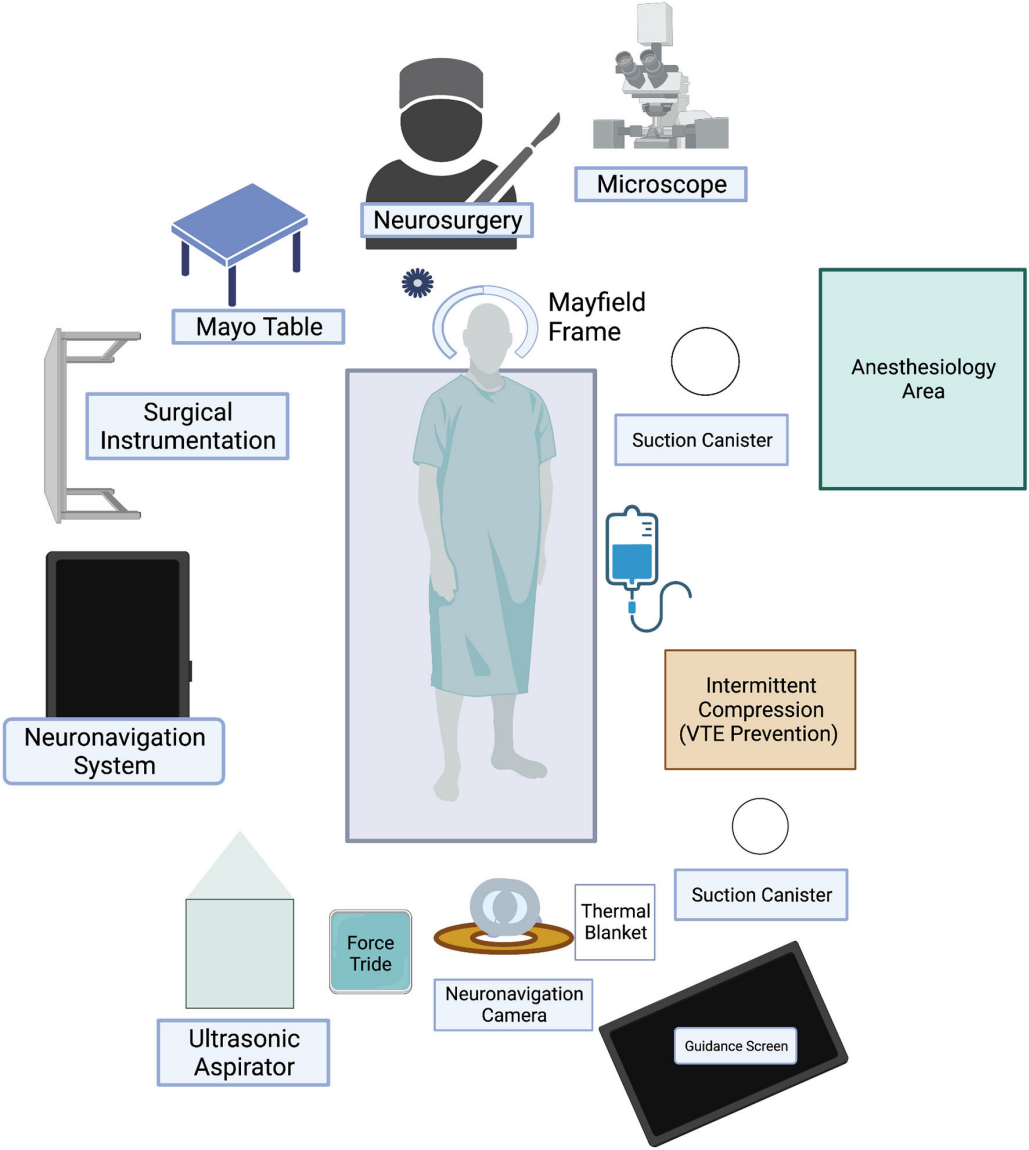
Microscope-based AR setup.

All steps for setting up AR for meningioma resection do not change compared to other brain lesions. The information injected depends on the capacity of the surgical planning software to integrate not only vessels but also diffusion tensor imaging or other remarkable neuroimaging data. The HUDs of the microscope are used for AR support. Injection of information for AR interface requires advanced processing from 3D reconstruction software. Checking the calibration of AR is performed by centering the microscope above the divot of the registration array. Thus, the optical outline and the AR visualization of the reference array can be adjusted, and the 3D reconstructed objects can also be visualized (preferably semitransparent to avoid obstruction of visual field visualization) (9). All operating room setup is demonstrated in Figure 1.

### 2.2. Reports of AR for meningioma resection

The integration of AR for tumor resection, including meningiomas resection, and mainly skull-base located meningiomas, has been pivotal for developing the technique (13). In 2010, Low et al. (14) presented the first cohort evaluating AR for the planning and navigating parasagittal, falcine, and convexity meningiomas. In this cohort, which included five patients, gross-total resection was achieved in 80% of the cases, and all patients returned to normal activities without complications. In this work, the authors highlight the use of technology to gain a better spatial appreciation, particularly around the venous anatomy. In addition, some concerns regarding the accuracy of the technique were also addressed (14). Later, in 2020, Lave et al. (11) published the first systematic review involving AR in intracranial meningiomas. The authors discussed eight studies, accounting for 20 parasagittal, falcine, and skull base meningiomas cases. The results showed that AR is beneficial for treating skull-base pathologies due to its visuospatial advantage, avoiding vascular injuries due to the rich anatomy landmarks in the skull-base, and enhancing the accuracy of the technique (11). Later, Pojskic et al. (9) presented a more extensive cross-sectional study including 39 patients, evaluating specifically skull-base meningioma resection. In this cohort, GTR was achieved in 67%, which is compatible with previous reports for meningioma resection in this location (without using AR) with rates of 63% for GTR (15). The authors also highlight the importance of including anatomical landmarks to increase the accuracy of the AR reconstruction (9).

The field also advanced toward minimally invasive surgeries. Jean et al. reported a video article using AR templates for a minimally invasive transorbital approach for intradural tumors, including meningiomas (16). This publication explored new utilities of the technique for more complex approaches with positive results; here, the authors highlighted the utility of anatomical landmarks (particularly bony landmarks) and an appropriate template to guide the drill toward the target (16).

### 2.3. Evolution of AR applied to meningioma resection

Oncologic neurosurgery is a highly complex field that has evolved by integrating various technologies to provide better patient outcomes. The first prototype of AR was designed in the 1960s, but only in the 1990s and 2000's that the interest in this technology increased, particularly in the medical field (17, 18). In the beginning, the use of AR was based on studies in other areas, such as the use of HUDs in maxillofacial surgery or pointer devices in stereotactic neurosurgery. It was not until 1999 that the first records of the use of AR in microscope-assisted interventions were published (19). AR has been extensively utilized across various neurosurgical subspecialties, particularly in enhancing the safety and precision of tumor resections (20). In 2010, the Dextroscope, an AR device, was described for the rapid and accurate resection of meningiomas (14). Since then, it has sought to update its applications with the integration of other devices such as tablets (21), HUDs/HMDs (22, 23), smartphones (24), and the integration of HUDs into the microscope (12). Finally, in 2021, Montemurro et al. (25) tested the precision of a wearable augmented reality platform (Video and Optical See-Through Augmented Reality Surgical System-VOSTAR) for parasagittal and convexity in plaque meningiomas bone-flap performance using a patient-specific 3D-printed model simulating a case. The researchers found that with AR, the simulation of the bone flap, presented an error of less than ±1 mm, also improving the depth-perception of the scene. These findings suggest the potential of AR to increase precision (25). All these technological advances have demonstrated considerable utility in different domains, such as preoperative planning, intraoperative navigation through real-time feedback, and education (20).

### 2.4. Current limitations and learned lessons

Intraoperative AR in micro neurosurgery depends on integrating the neuroimaging processing software into the hardware of both the microscope and the neuronavigation system. Currently, few systems with integrated HUDs allow this integration and adequate projection of images. The cost of these devices is considerably high, which undoubtedly poses a clear access limitation, even more so without demonstrating their actual usefulness. Regarding the operating room setup, the times can initially be increased up to ~30 min in the first cases, and progressively reduced to ~12 min. In order to improve workflow, the same surgical team must be trained in its implementation. The quality of the projected images will depend on the quality of their acquisition. To maximize the benefits of AR, we always suggest supplementing with other imaging modalities. We have always performed image fusion with CT and time-of-fly (TOF) magnetic resonance imaging for skull base lesions, achieving adequate vascular reconstructions. During the AR projection, the surgeon must adapt to the image overlay. However, the system allows easy removal of the projection. Thus, the surgeon can determine when the projected image will be most helpful.

**Figure 2:**
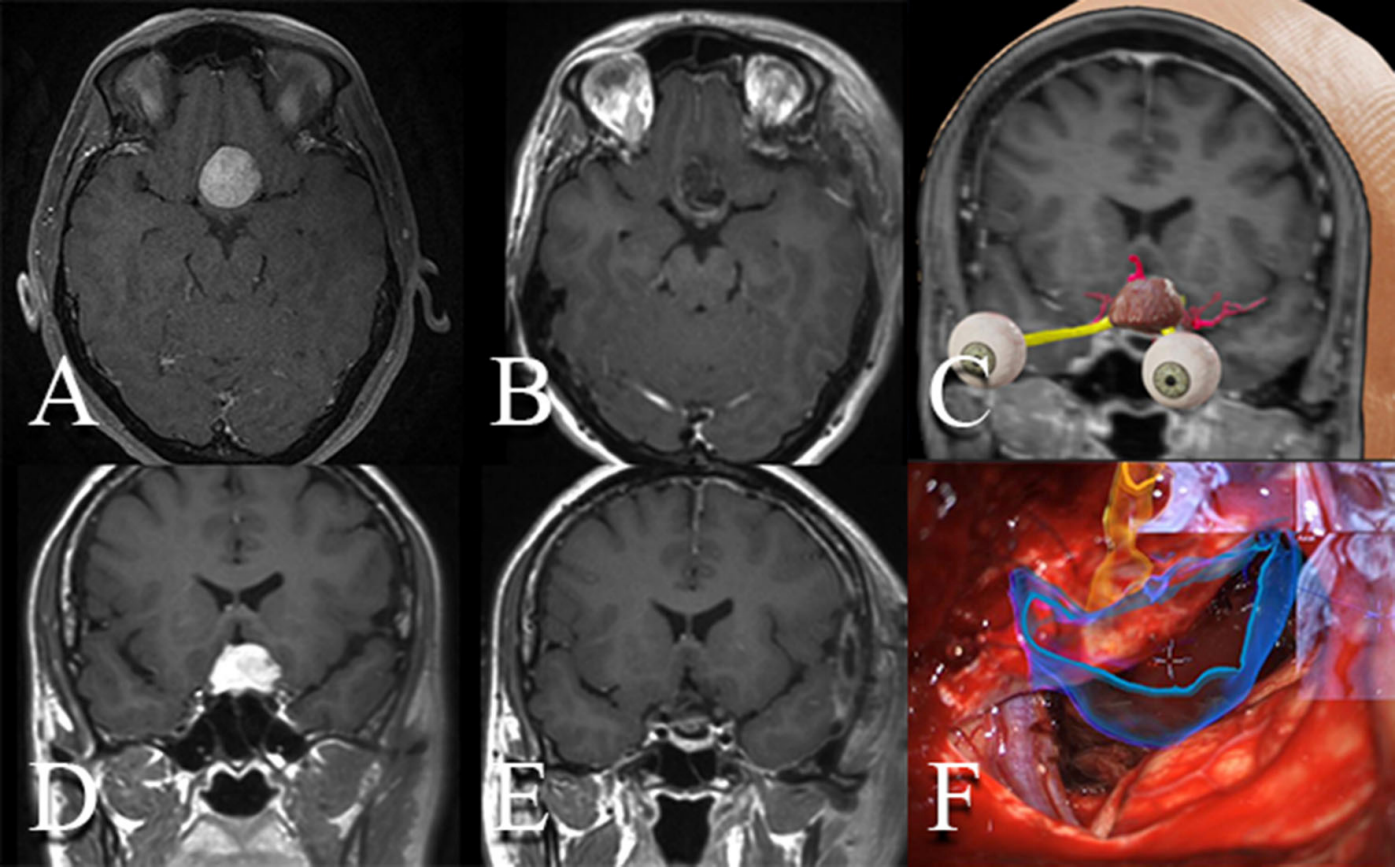
Resection of tuberculum sellae meningioma aided by intraoperative microscope integrated augmented reality. **(A, D)** Pre- and **(B, E)** postoperative enhanced MRI demonstrating a gross-total resection of a tuberculum sellae meningioma. **(C)** 3D reconstruction of the tumor, optic nerves, and arteries. **(F)** Intraoperative microsurgical picture using the microscope with integrated head-up displays. Volumetric 3D reconstruction of the tumor as well as of the optic apparatus are observed and displayed over the stereoscopic view of the microscope.

The position of the microscope is one of the most relevant references for proper navigation, and the microscope optics determines the depth of the focus point (focal distance). This is a pivotal aspect, given the adjustments required during surgery that may modify the projected image. The images of AR are projected from the neuronavigation system and have the same limitations as this technology. We also recommend looking for submillimeter precision during neuronavigation registration as much as possible to improve AR accuracy, as the information of regular volumetric CT/MR images and 3D segmentation reconstructions will be integrated into one single microscopical surgical view (Figure 2). It is important to remark that brain shift may be present, especially for intraparenchymal tumors and, to a lesser degree, for extra-axial skull base lesions. AR and its intraoperative use have become a tool that optimizes all available intraoperative imaging systems. It has allowed us to know the most precise location of structures not visible in the surgical field and the safe boundaries for surgical resection.

### 2.5. Learning curve and beginning process

Neurosurgeons face the challenge of learning, planning, and performing increasingly complex surgical procedures with a narrow window for error (26). AR has demonstrated the potential to increase accuracy in procedures like external ventricular drain (EVD) positioning (27, 28), percutaneous rhizotomy for trigeminal neuralgia (29, 30), and white matter tract dissection (31, 32). Starting with EVD placement, Van Gestel et al. used a cohort of sixteen medical students. After receiving a short introduction, the participants were randomly allocated to either the freehand or AR group. Each student had to perform at least four drains in a phantom-based head before receiving a standardized training session and four drains after the training. Results showed that, compared to the freehand technique, AR guidance for EVD placement yielded a higher outcome accuracy and quality in untrained individuals (27). A finding reinforced by Rossito et al. (33) in a cohort of 21 students and eight residents. Later, Bounajem et al. (28) showed in cadaveric models with individuals with different levels of training (medical students, residents, and attendings) that accuracy was improved with AR use at various levels depending on the training experience. AR has also been used in complex skull base cases. One example is the management of trigeminal neuralgia, demonstrated by Berger et al. (29), who showed that AR might enhance the learning curve, decreasing the time and radiation exposure in a percutaneous rhizotomy. These findings show different levels of complexity, where accuracy increased, regardless of the level of training, still with room for improvement in various interventions. The information for meningioma resection is scarce, further investigation on training with 3D non-biological models with the reconstruction of vessels and nerves may lead us to a better understanding of the curve before using it in the OR.

## 3. Discussion

Despite being a benign tumor in most cases, meningiomas remain a neurosurgical challenge, given their close relationships to crucial neurovascular structures, especially for those in the cranial base (34). Multiple efforts in designing strategies to prevent intraoperative injuries and obtain successful outcomes have been performed. The continuous development of tools like the intraoperative electrophysiological (35) and vascular monitoring (36) as well as image-guided systems (5, 9) have dramatically changed surgical approaches for safer resection. The introduction of virtual reality (37, 38) and AR (9, 39) has facilitated the 3D understanding of neurovascular anatomy and tumor boundaries. AR provides an excellent anatomical understanding of the pathology and surrounding structures (8), a feature that may help surgeons to facilitate tumor dissection and awareness of the location of vessels like the internal carotid arteries for tuberculum sellae meningiomas (16), superior sagittal sinus for parasagittal meningiomas (14), or vertebral and basilar arteries for petroclival, and foramen magnum meningiomas (39). A special emphasis must be done for the previously mentioned petroclival meningiomas, that due to their location are in closer relationship with multiple neurovascular structures (including the Internal Carotid Artery). In this case, AR is valuable alternative (39).

The literature and evidence remain scarce, and intraoperative AR indications remain in research.

Depuration of indications and real pros and cons are necessary to understand the benefits of its use. For now, it is mandatory to understand the basic principles of AR, introduce technology cautiously, given the progressive learning curve, and be aware of the limitations and possible errors associated with new technologies. At some point, the advancements in artificial intelligence may focus on AR into real benefits for a safer procedure. In the meantime, research is necessary, and a comparison of this technology with other image-guided tools is also necessary. AR and other technologies will never replace knowledge of microsurgical anatomy, nor the surgical skills required for adequate resection (de-vascularization, detachment, de-bulking, dissection) (34). Still, they may guide the surgeon into the anatomy of each patient in an individual case-by-case manner. Apparent benefits are focused on awareness of neurovascular structures (especially for cases with impaired anatomy like reoperations), improving magnification and awareness of tumor boundaries to prevent brain retraction, and a constant visualization of the surgical planning in the surgical field. For residents and trainees, AR provides an important approach to difficult cases. As suggested by Montemurro et al. (25), using AR for training, and surgical planning, in cadaveric or 3D printed models is a potential skill that can improve the performance, and have a positive impact in the learning curves. Other possible benefits would be the capacity to provide intraoperative information on the tumoral texture and consistency, and including vital signs and important data in real time (18); however, this remains an exciting research gap. The next step may be based on mixed reality, integrating different scenarios during the procedure, e.g., simulating brain retraction to predict adequate tumor exposure during surgery, like those proposed for intelligent vehicles (40). Integrating robotic minimally invasive surgery and constant intraoperative updates of the neuronavigation while using high-resolution cameras (40) and ultrasound (41) will provide real-time information without using intraoperative MRI scanners or other robust and more expensive systems.

## 4. Conclusions

Augmented reality (AR) integration in neurosurgery has shown promise in enhancing surgeons' awareness of critical neurovascular structures, facilitating tumor dissection, and improving overall surgical outcomes. While the literature on intraoperative AR remains limited, ongoing research has shown, along with other emerging technologies, that it can serve as a valuable tool to guide surgeons on an individual, case-by-case basis. There will always be a need to improve meningioma surgical results, and as advancements in artificial intelligence continue, integrating AR and mixed reality scenarios into neurosurgical procedures holds the promise of safer and more effective meningioma resections.

## Author contributions

DG: Conceptualization, Formal analysis, Methodology, Supervision, Writing—original draft, Writing—review & editing. EO-R: Conceptualization, Data curation, Investigation, Methodology, Writing—original draft, Writing—review & editing. AR-S: Conceptualization, Investigation, Methodology, Writing—original draft, Writing—review & editing. LF: Formal analysis, Software, Visualization, Writing—original draft, Writing—review & editing. MV-O: Investigation, Software, Writing—original draft, Writing—review & editing. JR: Conceptualization, Data curation, Investigation, Writing—review & editing. JM: Investigation, Supervision, Validation, Writing—review & editing. FH: Validation, Writing—original draft, Writing—review & editing.

## References

[1] Azuma RT. A survey of augmented reality. Pres Teleoper Virt Environ. (1997) 6:355–85. 10.1162/pres.1997.6.4.355

[2] Navab N Martin-Gomez A Seibold M Sommersperger M Song T Winkler A. Medical augmented reality: definition, principle components, domain modeling, and design-development-validation process. J Imaging. (2022) 9:1–4. 10.3390/jimaging9010004 36662102 PMC9866223

[3] Kyaw BM Saxena N Posadzki P Vseteckova J Nikolaou CK George PP. Virtual reality for health professions education: systematic review and meta-analysis by the digital health education collaboration. J Med Internet Res. (2019) 21:e12959. 10.2196/12959 30668519 PMC6362387

[4] Gerup J Soerensen CB Dieckmann P. Augmented reality and mixed reality for healthcare education beyond surgery: an integrative review. Int J Med Educ. (2020) 11:1–18. 10.5116/ijme.5e01.eb1a 31955150 PMC7246121

[5] Silva D Belsuzarri T Barnett GH. Image-guided surgery for meningioma. Handb Clin Neurol. (2020) 170:201–7. 10.1016/B978-0-12-822198-3.00040-9 32586491

[6] Akyuz ME Kadioglu HH. Application of neuronavigation system in intracranial meningioma surgery: a retrospective analysis of 75 cases. Cir Cir. (2022) 90:92–7. 10.24875/CIRU.22000201 36480746

[7] Carl B Bopp M Voellger B Sass B Nimsky C. Augmented reality in transsphenoidal surgery. World Neurosurg. (2019) 125:e873–e83. 10.1016/j.wneu.2019.01.202 30763743

[8] Luzzi S Giotta Lucifero A Martinelli A Maestro MD Savioli G Simoncelli A. Supratentorial high-grade gliomas: maximal safe anatomical resection guided by augmented reality high-definition fiber tractography and fluorescein. Neurosurg Focus. (2021) 51:E5. 10.3171/2021.5.FOCUS21185 34333470

[9] Pojskic M Bopp MHA Sabeta B Carl B Nimsky C. Microscope-based augmented reality with intraoperative computed tomography-based navigation for resection of skull base meningiomas in consecutive series of 39 patients. Cancers. (2022) 14:302. 10.3390/cancers14092302 35565431 PMC9101634

[10] Roethe AL Rosler J Misch M Vajkoczy P Picht T. Augmented reality visualization in brain lesions: a prospective randomized controlled evaluation of its potential and current limitations in navigated microneurosurgery. Acta Neurochir. (2022) 164:3–14. 10.1007/s00701-021-05045-1 34904183 PMC8761141

[11] Lave A Meling TR Schaller K Corniola MV. Augmented reality in intracranial meningioma surgery: report of a case and systematic review. J Neurosurg Sci. (2020) 64:369–76. 10.23736/S0390-5616.20.04945-0 32347678

[12] Jean WC. Mini-pterional craniotomy and extradural clinoidectomy for clinoid meningioma: optimization of exposure using augmented reality template: 2-dimensional operative video. Oper Neurosurg. (2020) 19:E610. 10.1093/ons/opaa238 32720680

[13] Cho J Rahimpour S Cutler A Goodwin CR Lad SP Codd P. Enhancing reality: a systematic review of augmented reality in neuronavigation and education. World Neurosurg. (2020) 139:186–95. 10.1016/j.wneu.2020.04.043 32311561

[14] Low D Lee CK Dip LL Ng WH Ang BT Ng I. Augmented reality neurosurgical planning and navigation for surgical excision of parasagittal, falcine and convexity meningiomas. Br J Neurosurg. (2010) 24:69–74. 10.3109/02688690903506093 20158356

[15] Meling TR Da Broi M Scheie D Helseth E. Meningiomas: skull base versus non-skull base. Neurosurg Rev. (2019) 42:163–73. 10.1007/s10143-018-0976-7 29627874

[16] Jean WC Sack KD Tsen AR. Augmented-reality template guided transorbital approach for intradural tumors. Neurosurg Focus Video. (2022) 6:V3. 10.3171/2021.10.FOCVID21172 36284590 PMC9555352

[17] Jean WC Britz GW DiMeco F Elmi-Terander A McIntyre C. Introduction. Virtual and augmented reality in neurosurgery: a timeline. Neurosurg Focus. (2021) 51:E1. 10.3171/2021.5.FOCUS21313 34333485

[18] Boaro A Moscolo F Feletti A Polizzi GMV Nunes S Siddi F. Visualization, navigation, augmentation. The ever-changing perspective of the neurosurgeon. Brain Spine. (2022) 2:100926. 10.1016/j.bas.2022.100926 36248169 PMC9560703

[19] Wagner A Ploder O Enislidis G Truppe M Ewers R. Virtual image guided navigation in tumor surgery–technical innovation. J Craniomaxillofac Surg. (1995) 23:217–3. 10.1016/S1010-5182(05)80155-6 8530700

[20] Cannizzaro D Zaed I Safa A Jelmoni AJM Composto A Bisoglio A. Augmented reality in neurosurgery, state of art and future projections. A systematic review. Front Surg. (2022) 9:864792. 10.3389/fsurg.2022.864792 35360432 PMC8961734

[21] Watanabe E Satoh M Konno T Hirai M Yamaguchi T. The trans-visible navigator: a see-through neuronavigation system using augmented reality. World Neurosurg. (2016) 87:399–405. 10.1016/j.wneu.2015.11.084 26732958

[22] Mascitelli JR Schlachter L Chartrain AG Oemke H Gilligan J Costa AB. Navigation-linked heads-up display in intracranial surgery: early experience. Oper Neurosurg. (2018) 15:184–93. 10.1093/ons/opx205 29040677 PMC6047456

[23] Fick T van Doormaal JAM Tosic L van Zoest RJ Meulstee JW Hoving EW. Fully automatic brain tumor segmentation for 3D evaluation in augmented reality. Neurosurg Focus. (2021) 51:E14. 10.3171/2021.5.FOCUS21200 34333477

[24] Chen JG Han KW Zhang DF Li ZX Li YM Hou LJ. Presurgical planning for supratentorial lesions with free slicer software and sina app. World Neurosurg. (2017) 106:193–7. 10.1016/j.wneu.2017.06.146 28673889

[25] Montemurro N Condino S Cattari N D'Amato R Ferrari V Cutolo F. Augmented reality-assisted craniotomy for parasagittal and convexity en plaque meningiomas and custom-made cranio-plasty: a preliminary laboratory report. Int J Environ Res Pub Health. (2021) 18:955. 10.3390/ijerph18199955 34639256 PMC8507881

[26] Chan S Conti F Salisbury K Blevins NH. Virtual reality simulation in neurosurgery: technologies and evolution. Neurosurgery. (2013) 72:154–64. 10.1227/NEU.0b013e3182750d26 23254804

[27] Van Gestel F Frantz T Vannerom C Verhellen A Gallagher AG Elprama SA. The effect of augmented reality on the accuracy and learning curve of external ventricular drain placement. Neurosurg Focus. (2021) 51:E8. 10.3171/2021.5.FOCUS21215 34333479

[28] Bounajem MT Cameron B Sorensen K Parr R Gibby W Prashant G. Improved accuracy and lowered learning curve of ventricular targeting using augmented reality-phantom and cadaveric model testing. Neurosurgery. (2023) 92:884–91. 10.1227/neu.0000000000002293 36562619

[29] Berger A Choudhry OJ Kondziolka D. Augmented reality-assisted percutaneous rhizotomy for trigeminal neuralgia. Oper Neurosurg. (2023) 24:665–9. 10.1227/ons.0000000000000661 36815787

[30] Rau A Roelz R Urbach H Coenen VA Demerath T Reinacher PC. Application of augmented reality in percutaneous procedures-rhizotomy of the gasserian ganglion. Oper Neurosurg. (2021) 21:160–4. 10.1093/ons/opab155 34098574 PMC8555421

[31] Aydin SO Barut O Yilmaz MO Sahin B Akyoldas G Akgun MY. Use of 3-dimensional modeling and augmented/virtual reality applications in microsurgical neuroanatomy training. Oper Neurosurg. (2023) 24:318–23. 10.1227/ons.0000000000000524 36701556

[32] Ille S Ohlerth AK Colle D Colle H Dragoy O Goodden J. Augmented reality for the virtual dissection of white matter pathways. Acta Neurochir. (2021) 163:895–903. 10.1007/s00701-020-04545-w 33026532 PMC7966623

[33] Rossitto CP Odland IC Oemke H Cruz D Kalagara R Schupper AJ. External ventricular drain training in medical students improves procedural accuracy and attitudes toward virtual reality. World Neurosurg. (2023) 175:e1246–e54. 10.1016/j.wneu.2023.04.108 37149087

[34] Ius T Tel A Minniti G Somma T Solari D Longhi M. Advances in multidisciplinary management of skull base meningiomas. Cancers. (2021) 13:664. 10.3390/cancers13112664 34071391 PMC8198762

[35] Shkarubo AN Chernov IV Ogurtsova AA Chernov VE Borisov OV Koval KV. Cranial nerve monitoring in endoscopic endonasal surgery of skull base tumors (observing of 23 cases). Chin Neurosurg J. (2018) 4:38. 10.1186/s41016-018-0146-3 32922898 PMC7398298

[36] Wiedmann M Lashkarivand A Berg-Johnsen J Dahlberg D. How I do it: endoscopic endonasal resection of tuberculum sellae meningioma. Acta Neurochir. (2021) 163:2193–7. 10.1007/s00701-021-04784-5 33665730 PMC8270812

[37] Jean WC Wang CP Rios-Vicil CI. Transsulcal, transchoroidal approach for resection of posterior clinoid meningioma with virtual reality demonstration: 2-dimensional operative video. Oper Neurosurg. (2022) 23:e286. 10.1227/ons.0000000000000323 36001759

[38] Jean WC Yang Y Srivastava A Tai AX Herur-Raman A Kim HJ. Study of comparative surgical exposure to the petroclival region using patient-specific, petroclival meningioma virtual reality models. Neurosurg Focus. (2021) 51:E13. 10.3171/2021.5.FOCUS201036 34333476

[39] Matsoukas S Oemke H Lopez LS Gilligan J Tabani H Bederson JB. Suboccipital craniectomy for an anterior foramen magnum meningioma-optimization of resection using intraoperative augmented reality: 2-dimensional operative video. Oper Neurosurg. (2022) 23:e321. 10.1227/ons.0000000000000373 36103323

[40] Li Y Cai Y Malekian R Wang H Sotelo MA Li Z. Creating navigation map in semi-open scenarios for intelligent vehicle localization using multi-sensor fusion. Expert Syst Appl. (2021) 184:115543. 10.1016/j.eswa.2021.115543

[41] Sass B Zivkovic D Pojskic M Nimsky C Bopp MHA. Navigated intraoperative 3D ultrasound in glioblastoma surgery: analysis of imaging features and impact on extent of resection. Front Neurosci. (2022) 16:883584. 10.3389/fnins.2022.883584 35615280 PMC9124826

